# Histeria

**Published:** 1890-04-12

**Authors:** 


### HYSTERIA.

We find in the last number to hand of the *American Lancet* a lecture by Dr. H. C. Wood on the management and treatment of hysteria and neurasthenia. Dr. Wood defines the latter term as meaning "lack of nerve power," and he says any expenditure of force may cause it. "Individuals vary as much in their nerve power as they do in their moneyed capital." There are many families whose members are neurasthenics from birth . . . . living is a labour, and a person who lives beyond his means soon sinks . . . . the same is true of nerve capital. In hysteria, the fundamental condition is one of weakness and lack of control of inhibition, "The nerve power being wanted in the patient, you must bring your nerve power to bear on the case." Dr. Wood illustrates his meaning by mentioning the case of a young woman engaged to be married, who had hysterical vomiting. The fiancé was induced to declare he would not marry the girl unless she were cured of her vomiting. "The young man was firm, and in a few weeks she vomited no more." In taking care of a hysterical case you must have absolute control over her, and sympathising friends must be kept away. "If a hysterical patient wakes in the morning, and says the light hurts her eyes and makes her worse, the nurse must pay no attention to her but open the window widely, and tell the patient that the doctor ordered plenty of light." If a nurse sympathizes with a patient, the latter will never get well. Sometimes it may be necessary to change the nurse, "as human nature cannot resist the affectionate sorrow of an hysterical woman. No nurse ought, therefore, to feel hurt if she is dismissed before a case is cured." Now we are all aware that sympathy very often is misplaced with hysterical people. Hysterical persons, however, should never be treated unkindly or roughly, for it does not follow that because a person is hysterical such person may not have some other disease. A form of nervous disturbance, much resembling hysteria but of very serious import, sometimes attends Bright's disease. And Dr. Buzzard has recently pointed out that hysteria may depend on sclerosis of the spinal marrow. Although hysterical patients cannot altogether avoid their attacks, they can, to a certain extent, guard themselves against the seizures; and this they should be made to understand they are expected to do.

